# Lightning‐Fast Convective Outlooks: Predicting Severe Convective Environments With Global AI‐Based Weather Models

**DOI:** 10.1029/2024GL110960

**Published:** 2024-11-21

**Authors:** Monika Feldmann, Tom Beucler, Milton Gomez, Olivia Martius

**Affiliations:** ^1^ Institute of Geography Oeschger Centre for Climate Change Research University of Bern Bern Switzerland; ^2^ Faculty of Geosciences and Environment Expertise Center for Climate Extremes University of Lausanne Lausanne Switzerland

**Keywords:** severe convection, convective environments, artificial intelligence, forecasting, medium‐range

## Abstract

Severe convective storms are among the most dangerous weather phenomena and accurate forecasts mitigate their impacts. The recently released suite of AI‐based weather models produces medium‐range forecasts within seconds, with a skill similar to state‐of‐the‐art operational forecasts for variables on single levels. However, predicting severe thunderstorm environments requires accurate combinations of dynamic and thermodynamic variables and the vertical structure of the atmosphere. Advancing the assessment of AI‐models toward process‐based evaluations lays the foundation for hazard‐driven applications. We assess the forecast skill of the top‐performing AI‐models GraphCast, Pangu‐Weather and FourCastNet for convective parameters at lead‐times up to 10 days against reanalysis and ECMWF's operational numerical weather prediction model IFS. In a case study and seasonal analyses, we see the best performance by GraphCast and Pangu‐Weather: these models match or even exceed the performance of IFS for instability and shear. This opens opportunities for fast and inexpensive predictions of severe weather environments.

## Key Points

- AI‐based global weather models produce forecasts with sufficient accuracy to derive instability and shear metrics skillfully
- The best AI‐based weather models are capable of competing with state‐of‐the‐art numerical weather predictions of instability and shear
- This is a major step toward computationally inexpensive and fast convective outlooks

## 1 Introduction

Artificial intelligence (AI)‐based deterministic forecasts are revolutionizing the landscape of medium‐range weather forecasting (Bouallègue et al., 2024; Ebert‐Uphoff & Hilburn, 2023). Among the models released in the past year are FourCastNet (Bonev et al., 2023), GraphCast (Lam et al., 2023) and Pangu‐Weather (Bi et al., 2023). They are capable of forecasting the main atmospheric state variables on various pressure levels within seconds (on GPU, Wong, 2023), with a skill similar to the deterministic IFS model (Integrated Forecasting System, Bougeault et al., 2010, operational version of IFS) of the ECMWF (European Centre for Medium‐range Weather Forecasts). AI‐based weather predictions are rapidly revolutionizing weather forecasting (Beucler, Koch, et al., 2024; Bouallègue et al., 2024; Brunet et al., 2023; Govett et al., 2024; McGovern et al., 2024; Olivetti & Messori, 2024a) with scientists and forecasting centers calling for action to scrutinize AI‐model performance from various perspectives, going beyond simply verifying the atmospheric state (Bonavita, 2024; Ebert‐Uphoff & Hilburn, 2023; Jeppesen, 2024). First studies investigate the robustness of forecasts for extremes (Charlton‐Perez et al., 2024; Olivetti & Messori, 2024b) and model behavior in idealized experiments (Hakim & Masanam, 2024).

We focus on the specific, application‐oriented task of accurately predicting atmospheric profiles in convective environments. The skillful prediction of convective environments requires high accuracy in the vertical profile and the simultaneous, correct prediction of both thermodynamic (temperature and humidity) and kinematic components (vertical wind shear). Convective storms require instability, governed by the vertical temperature and moisture profile (e.g., Houze, 2014; Trapp, 2013). Severity is modulated by wind shear, emphasizing the importance of the wind profile (Houze, 2014; Trapp, 2013). By combining instability and wind shear, severe convective environments, that are related to particularly hazardous and impactful thunderstorms, can be identified (e.g., H. E. Brooks et al., 2003; Kunz, 2007; Taszarek et al., 2020).

Severe thunderstorms are among the most hazardous weather phenomena and caused substantial losses worldwide in recent years (e.g., Bowen et al., 2024). Severe thunderstorms can produce large hail and severe wind gusts, along with torrential rain, lightning strikes, and tornadoes (Houze, 2014; Markowski & Richardson, 2010).

Global AI‐models at medium‐range lead‐times currently provide weather information on a 0.25° grid. These models can hence not explicitly forecast severe storms. Therefore we focus on verifying pre‐convective environments, characterized by mesoscale patterns of instability and wind shear. These environments provide information on the propensity of the atmosphere to produce severe storms (e.g., H. E. Brooks, 2013; Taszarek et al., 2020, 2021) and are used to forecast severe convection (Battaglioli et al., 2023; H. Brooks et al., 2011; Kunz, 2007). Accurate and inexpensive forecasts of mesoscale convective environments enable the timely assessment of severe convective outlooks for hazardous weather warnings.

## 2 Data

We focus our analysis on the year 2020, which is established as a reference year in the benchmark data set “Weatherbench2” (Rasp et al., 2024) and was not used in training AI‐models. We evaluate the three AI‐models Pangu‐Weather (Bi et al., 2023), GraphCast (Lam et al., 2023), and FourCastNet (Bonev et al., 2023). We selected models with publicly accessible code and a stable version. Other AI‐based global models have been developed (AIFS, FuXi, Aurora, and ClimaX; Bodnar et al., 2024; Chen et al., 2023; Lang et al., 2024; Nguyen et al., 2023), but do not meet our evaluation requirements. The models are compared with the performance of the deterministic IFS‐HRES (Haiden et al., 2019, IFS cycle 46r1). The reference model IFS is the operational, physics‐based, numerical weather prediction model at ECMWF, used for global medium‐range forecasts (Bougeault et al., 2010; Thepaut & Courtier, 1991). The ERA‐5 reanalysis (Hersbach et al., 2020) serves as a ground truth. We obtain Pangu‐Weather, GraphCast, and IFS data through “Weatherbench2” (Rasp et al., 2024). FourCastNet forecasts are produced with the “ai‐models” code‐release of ECMWF (ECMWF, 2023).

In terms of architecture, Pangu‐Weather is a transformer model that uses a 3‐D visual earth transformer to encode the relative location on a sphere and additionally employs a hierarchical temporal aggregation, combining a 24 hr‐prediction model with a 6 hr‐prediction model (Bi et al., 2023). GraphCast is a graph neural network, which is particularly useful for spatially structured data, and uses successive 6 hr‐predictions (Lam et al., 2023). “FourCastNet v2 small” is a transformer model, employing spherical Fourier neural operators (SFNO) to account for the spatial encoding of a sphere (Bonev et al., 2023).

All data is gridded at a 0.25° resolution, and IFS’ native 0.125° resolution is regridded using the MIR method (Meteorological Interpolation and Regridding, Barratt, 2017). The forecasts are available twice daily at 00 hr UTC and 12 hr UTC up to 10 days lead‐time in 6‐hr increments. The AI‐models are initialized from both ERA‐5 and the IFS analysis. This reflects the conditions they were trained in (ERA‐5) compared to an operational prediction set‐up (IFS analysis). GraphCast has an additional operational version specifically tuned to being initialized with IFS data (GraphCast‐oper). IFS is initialized with the IFS analysis.

For the computation of convective parameters, we use the temperature (T), specific humidity (Q), geopotential, and horizontal wind on the surface and pressure levels listed in Table S1 in Supporting Information S1 (see Section 3.1). The models differ in the available pressure levels and in the moisture variable that they predict, with most models providing 13 pressure levels between 50 and 1,000 hPa and predicting Q. FourCastNet predicts relative humidity (RH), which needs to be converted to Q before deriving CAPE. An overview is provided in Table S1 in Supporting Information S1.

## 3 Methods

### 3.1 Deriving Convective Parameters

We focus on the following convective parameters: convective available potential energy (CAPE), deep‐layer shear (DLS), and a combined parameter of both called wmax‐shear (WMS). CAPE is derived with the implementation for most unstable CAPE found in WRF‐python (Ladwig, 2017), which launches a parcel profile above the surface at the height of highest equivalent potential temperature, using temperature, moisture, and geopotential at pressure levels, as well as surface elevation, pressure, and temperature. The relatively coarse vertical resolution reduces the accuracy of the CAPE calculation (Mensch, 2021; Wang et al., 2021). However, we apply the same procedure to all data sets, hence treating them equally. We thereby artificially reduce the number of vertical levels available in the IFS and ERA‐5 data. To approximate DLS, we compute the magnitude of the differential wind vector between the 10 m and the 500 hPa level. Further details on the computation of convective parameters are described in Text S1 in Supporting Information S1.

### 3.2 Forecast Evaluation

To evaluate the forecast quality, we use several different scores. We first compute the root mean square error (RMSE) and the bias, two standard scores. To account for displacement errors and avoid the so‐called “double penalty” (Gilleland, 2013; Gilleland et al., 2009), we additionally use the fractional skill score (FSS, Mittermaier, 2021) and the structure, amplitude, and location (SAL) score, which evaluates these three properties individually (Wernli et al., 2008, 2009). Additional information on the interpretation of the scores is provided in Table S2 in Supporting Information S1.

We compute the FSS for two thresholds for each variable (300 and 1000 J kg^−1^ CAPE; 300 and 500 m^2^ s^−2^ WMS, Taszarek et al., 2017), with a neighborhood size of 1°, oriented on the gradients between categories of convective outlooks. The SAL is computed for 300 J kg^−1^ CAPE, describing the convective environment. While the thresholds also include environments with only a moderate likelihood of convective storm formation, they serve as an estimated lower bound on severe convective storm occurrence.

## 4 Case Study: US Tornado Outbreak

### 4.1 Synoptic Situation

Throughout 12 and 13 April 2020, 141 tornadoes were reported in 10 states, ranging up to a strength of EF4 (Storm Prediction Center, 2020a, 2020b). With an estimated 450 Million US$ worth of property damage and 38 fatalities, this tornado outbreak was among the highest‐impact events in the southern states (Beddoes, 2020), contributing to 2020 having the highest severe convective storm damages in the past (Bevere & Weigel, 2021).

The tornado outbreak was characterized by an anomalously warm period in the preceding month (Copernicus Services, 2020), leading to high sea surface temperatures in the Gulf of Mexico. The convective period was induced by a deep trough moving across the US. In the warm sector, warm and moist air from the Gulf was advected over the Southeastern states. Simultaneously, the approaching trough was associated with high DLS, which is crucial for convective organization. The convective potential of the situation was well forecast, the storm prediction center issued the first warnings 4 days ahead (Dean & Dial, 2020).

**Figure 1:**
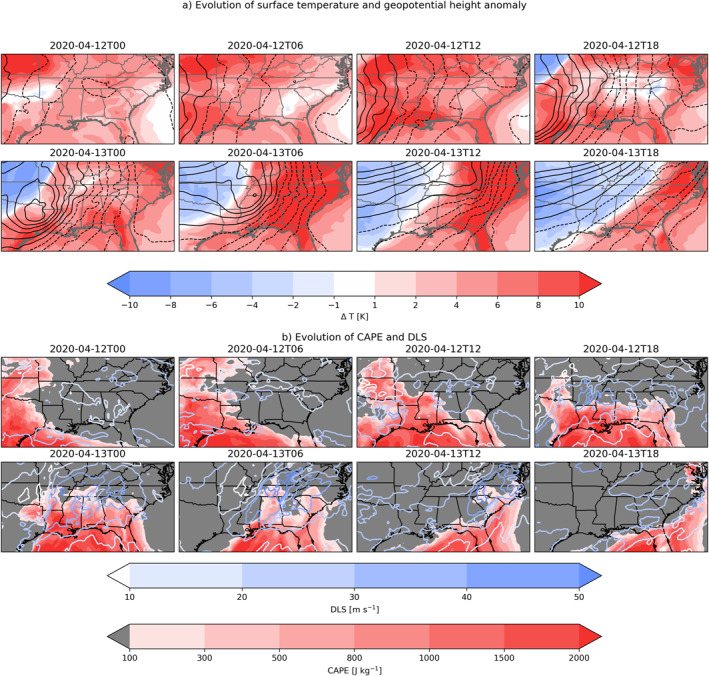
Synoptic situation in ERA‐5 on April 12 and 13 2020, showing a deep trough moving across the United States, with anomalously warm temperatures and higher CAPE values in the warm sector; (a) Climatological geopotential height anomaly and surface temperature anomaly, contours indicate positive (dashed) and negative (solid) anomalies of geopotential height in 20 m increments (b) pressure‐level‐derived CAPE and DLS, hatched area indicates where DLS > 20 m s^−1^.

While this event was most striking because of the large number of tornadoes, we focus on the convective environment characterized by CAPE and DLS. Figure 1 shows the evolution of pressure‐level CAPE, DLS, the 500 hPa geopotential height anomaly, and the surface temperature anomaly from ERA‐5 during the outbreak.

The in‐depth analysis of model forecast performance will focus on 12 April 2020 at 12:00 UTC, a few hours before the most intense storms occurred.

### 4.2 Forecasting CAPE and DLS

**Figure 2:**
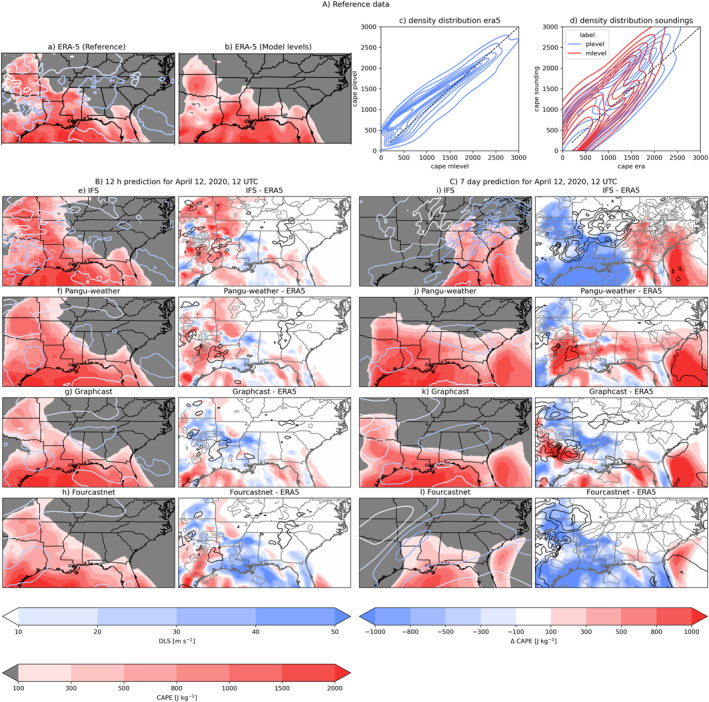
Forecast comparison of CAPE and DLS at 12 hr and 7 days lead‐time; (A) ERA‐5 data of CAPE and DLS on 12 April 2020 at 12 UTC; (a) CAPE and DLS from pressure levels, (b) CAPE from model levels, (c) density diagram of CAPE from pressure and model levels, (d) density diagram of CAPE from pressure and model levels against observed soundings. (B) 12 hr forecast of CAPE and DLS in comparison to ERA5 (e)–(h) (C) 7‐day forecast of CAPE and DLS in comparison to ERA5 (i–l); contours indicated positive (gray) and negative (black) areas of Δ DLS in 5 m s^−1^ increments.

We established CAPE from ERA‐5 pressure levels as the reference. However, to assess realistic convective outlooks, we additionally compare this to CAPE derived from model levels and soundings. Figure 2A shows a comparison of both (Figures 2a and 2b) and their density distribution over the entire case duration (Figure 2c). They correlate well ($R^{2}$ = 0.79), however, the distribution is narrowed, with an overestimation of low values and an underestimation of high values. When comparing to soundings (Figure 2d), any ERA‐5 derived CAPE generally tends to be underestimated with respect to the radio‐sounding. Diagrams of the observed soundings and ERA‐5‐modeled pseudo‐soundings are provided in Figure S1 in Supporting Information S1.

We first determine whether AI‐models overall produce realistic CAPE and DLS values at a short lead‐time of 12 hr. Figure 2B shows all models initialized with IFS analysis (Figures 2e–2h, absolute and difference to ERA‐5). All models produce CAPE values in the right order of magnitude with a spatial distribution of DLS that corresponds well to the reference. Nonetheless, we can see differences between the models. All AI‐model fields appear smoother than the reference data. IFS has the highest degree of spatial detail in both DLS and CAPE, while the AI‐models mostly capture the larger‐scale features. The high degree of detail in IFS, however, is penalized in the standard performance scores, as already a small displacement of features can lead to increases in RMSE (Figure 2e, RMSE‐IFS = 312 J kg^−1^, RMSE‐GraphCast‐oper = 271 J kg^−1^; for all scores see Table S4 in Supporting Information S1). FourCastNet‐oper underestimates CAPE in this case (Figure 2h). Of the AI‐models, GraphCast‐oper shows the closest match in terms of location and magnitude to ERA‐5 (Figure 2g).

We next inspect the lead‐time 168 hr/7 days (Figure 2C). At this longer lead‐time, IFS displaces the event eastwards (Figure 2i), however, the magnitude of CAPE values is correct. This leads to large absolute errors (RMSE‐IFS 829 J kg^−1^). Pangu‐Weather‐oper's forecast also suffers from displacement error, owed to a very different spatial structure of the event (Figure 2j, RMSE‐PanguWeather‐oper 570 J kg^−^^1^). GraphCast‐oper has the best agreement in terms of event structure and location (Figure 2k, RMSE‐GraphCast‐oper 520 J kg^−1^). FourCastNet‐oper strongly underestimates CAPE and also has the largest degree of smoothing (Figure 2l). It misses most land areas that experience high CAPE and DLS. Nonetheless, we highlight here, that all models capture an area of elevated CAPE values more than 6 days in advance, in co‐location with high DLS.

## 5 Seasonal and Regional Analyses

To investigate performance scores, we derive CAPE and WMS for North America, Europe, Argentina, and Australia and their respective convective seasons. CAPE and WMS are filtered by a land‐sea‐mask to focus on impact relevant areas. Table S3 in Supporting Information S1 summarizes the regions' extent and seasons. We then compute the lead‐time‐dependent scores for each model.

### 5.1 The North American Convective Season

**Figure 3:**
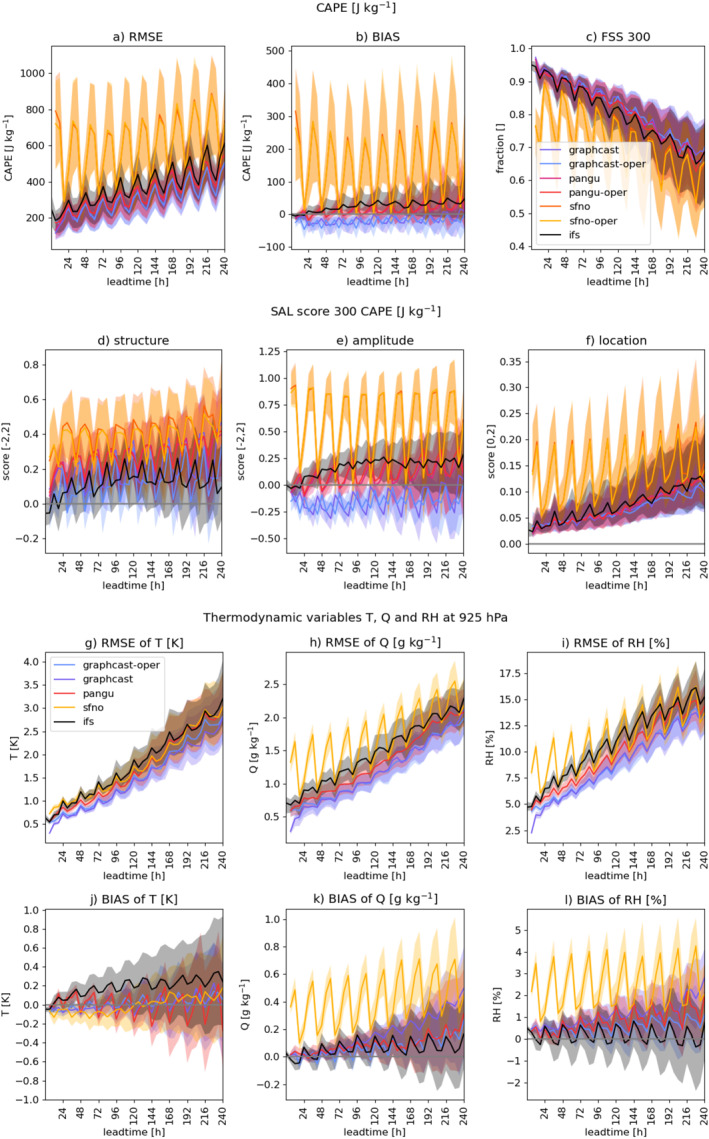
Seasonal evaluation of CAPE for North America at 00 UTC initialization shows Pangu‐Weather and GraphCast outperforming IFS, with a moisture bias impacting the performance of FourCastNet. Top row: (a) RMSE, (b) BIAS, (c) FSS_300_; Second row: SAL components for CAPE > 300 J kg^−1^; color legend in panel (c); Third row: RMSE of T, Q and RH at 925 hPa (g)–(i); Fourth row: BIAS (j–l); model legend in panel (g). Solid line depicts the median and shading the interquartile range.

Figure 3 depicts the lead‐time‐dependent scores throughout the entire convective season for the USA. We include here both the models initialized with ERA‐5 and with IFS analysis, initialized at 00 UTC.

The RMSE of CAPE (Figure 3a) shows close proximity of the scores of Pangu‐Weather, GraphCast, and IFS. They lie well within each other's interquartile range (IQR). FourCastNet is a notable exception, performing consistently worse than all other models. GraphCast and Pangu‐Weather tend to outperform IFS slightly. Since they are optimized on RMSE (albeit on different variables), we expect them to perform best in this metric. The BIAS (Figure 3b) shows more diversity, with IFS having an increasingly positive BIAS and Pangu‐Weather a quite stationary BIAS, centered on 0. GraphCast moderately underestimates CAPE, while FourCastNet strongly overestimates CAPE, contrasting with the underestimation in the case study. The seasonal analysis is dominated by the principal convective season in the Great Plains, whereas the case study focuses on a spring convective event in the Southeast. For the FSS_300_ (Figure 3c), GraphCast and Pangu‐Weather are slightly better than IFS at intermediate lead‐times. FourCastNet is consistently worse, largely lying outside of the IQR of the other models. In terms of SAL scores, the amplitude score mirrors the BIAS (Figures 3b and 3e). The positive structure score shows the smoother behavior of the AI‐models (Figure 3d). It is important to note, that structure, amplitude, and location are not wholly independent of one another, as strong biases also affect structure and location of objects. The location score shows the largest dependence on lead‐time, steadily increasing (Figure 3f). Overall IFS scores close to “0” in both structure and amplitude, while the location score shows the strongest deterioration over lead‐time.

A clear pattern visible in all models is a regular oscillation on a 24‐hr frequency, when separating the data into the 00 UTC (Figure 3) and 12 UTC (Figure S2 in Supporting Information S1) initializations. This is owed to the strong diurnal cycle of CAPE, which depends on the diurnal cycle of temperature (Lawson & Gallus Jr, 2016; Leon, 2018) and humidity. The models tend to perform better at night and in the early morning when CAPE is naturally low. The errors are higher during the day and in the evening when peak CAPE values occur. For Pangu‐Weather this additionally coincides with the change between the 6 hr‐ and 24 hr‐integration models, leading to a 24 hr cycle of skill even in combined initialization times (see Section 5.3).

**Figure 4:**
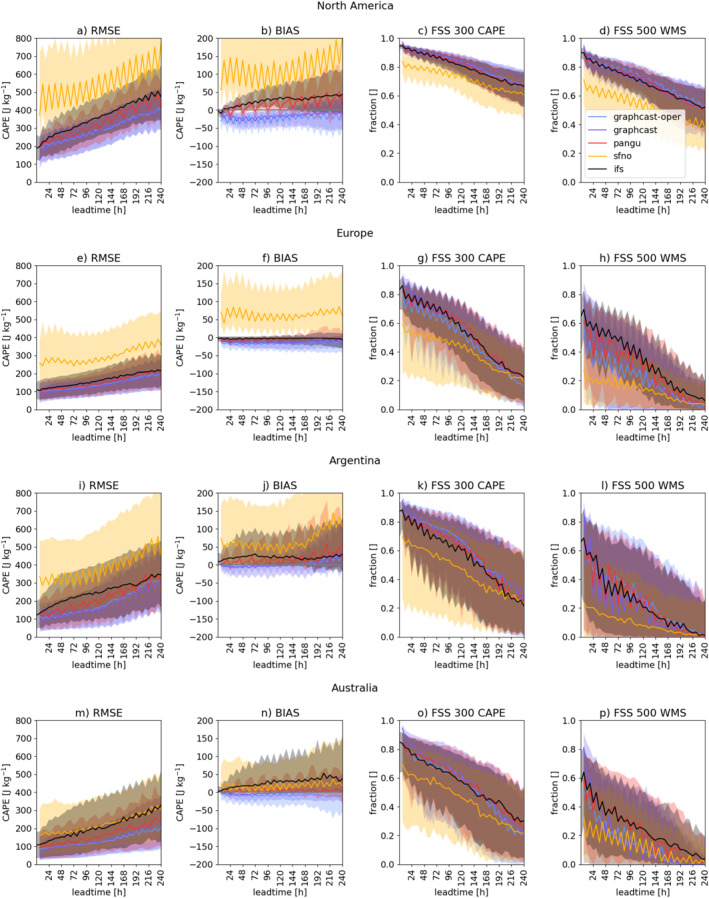
Seasonal evaluation of CAPE in North America (Apr–Aug, a–d), Europe (Apr–Sep, e–h), Argentina (Sep–Feb, i–l) and Australia (Sep–Feb, m–p) shows good performance of GraphCast and Pangu‐Weather; solid line depicts median of score, the IQR is shown in shading.

Given the similarity between the initializations with ERA5 and IFS analysis, we focus the next analyses on the initializations with IFS (previously flagged as “oper”), mimicking an operational context. Since GraphCast (initialized with ERA‐5) and GraphCast‐oper (initialized with IFS) are two different model versions, we maintain both.

### 5.2 The Impact of Thermodynamic Conversions

With FourCastNet being an outlier, one core difference is the prediction of RH instead of Q. Q is used most commonly, but due to the relatively strong boundedness, RH has been shown to generalize better in some machine learning applications (Beucler, Gentine, et al., 2024; Lin et al., 2024). This mostly applies to large domain shifts under climate change, where Q is no longer well‐constrained. At the weather timescale, Q can be assumed to be in a stable value range. To calculate CAPE, RH needs to be converted to Q. The conversion is dependent on the temperature and pressure, making this an additional processing step, where forecast errors can compound. To quantify this effect, we additionally compare all models' RMSE and BIAS for RH, Q, and T in Figure 3.

The RMSE (Figure 3g) and BIAS (Figure 3j) of T are quite similar across all models, indicating that the large differences in CAPE are not primarily owed to T. At short lead‐times FourCastNet performs only marginally worse. Looking at Q, it becomes evident that FourCastNet not only has a larger RMSE (Figure 3h) but also consistently overestimates Q (Figure 3k), which does not improve much at short lead‐times. For RH it becomes evident, that despite this being the natively predicted variable, FourCastNet also does not perform particularly well. The BIAS (Figure 3l) remains consistently positive. GraphCast‐oper and Pangu‐Weather consistently have a lower RMSE and BIAS (Figures 3i and 3l), despite RH being a converted variable in their output. Consequently, FourCastNet suffers from a double penalty ‐ the moderately good RH undergoes further conversion to Q before being integrated into the computation of CAPE. Moisture is a key component in determining the lifting condensation level, which strongly impacts the integral of CAPE, leading to large compounding errors.

### 5.3 Global Convective Hotspots

We next compare the models' performance in additional convectively active regions: Europe, Argentina, and Australia. Figure 4 depicts the RMSE, BIAS, and FSS_300_ for CAPE, and the FSS_300_ for WMS, computed on both the 00 UTC and 12 UTC initializations.

At first glance, the RMSE is highest in North America and Argentina (Figures 4a and 4i). This is partly due to the overall magnitude of CAPE values during the 2020 convective seasons; a value of 300 J kg^−1^ falls at the 84th and 86th percentiles of the CAPE distribution in these regions, compared to the 90th and 93rd percentiles in Australia and Europe, respectively. The magnitude of CAPE impacts the FSS_300_, as the FSS generally decreases as the threshold approaches the tail end of the overall value distribution. Hence it is consistent that the FSS_300_ is overall best in North America (Figure 4c).

FourCastNet generally performs worse and has a wider range of performance scores than the other models. This is primarily driven by the quality of its RH forecast. Australia is an exception, where FourCastNet achieves similar RMSE (Figure 4m) to the other models, albeit with a very large IQR. The lower RMSE of CAPE stems from a better prediction of RH in this region (figure not shown).

In all regions, GraphCast‐oper and GraphCast show the lowest RMSE of CAPE (Figures 4a, 4e, 4i, and 4m) and over most lead‐times the highest FSS_300_ (Figures 4c, 4g, 4k, and 4o), followed by Pangu‐Weather and IFS. Despite the small differences, the high consistency across multiple regions shows this as systematic behavior.

The FSS_300_ of CAPE and FSS_300_ of WMS show similar behavior, with the WMS achieving lower scores, being at a higher convective threshold. This indicates that skill is primarily driven by CAPE, which dominates the forecast‐error, while shear poses a less complicated forecast problem. Both FSS show very similar performance for GraphCast, Pangu‐Weather, and IFS. There is no consistent ranking as in the RMSE, highlighting the importance of considering multiple performance scores.

## 6 Discussion and Conclusion

Pangu‐Weather, GraphCast‐oper, and GraphCast are capable of producing realistic forecasts of the mesoscale convective environment at medium‐range lead‐times in a matter of minutes and perform similarly to IFS. FourCastNet has the strongest deficiencies, partially owed to the additional processing step from RH to Q. However, as Section 5.2 shows, forecasting errors in convective parameters are also owed to overall issues in forecasting moisture. With GraphCast and Pangu‐Weather providing reasonable RH values derived from Q, we conclude that RH is not necessarily a worse choice than Q, but rather that “FourCastNet v2 small” specifically has issues in forecasting moisture. The best choice in moisture variable depends on the application, as every thermodynamic conversion in a non‐physical model comes with additional uncertainty.

The evaluation of different regions shows the global capability of Pangu‐Weather and GraphCast. Despite regional performance differences, the ranking of models remains stable, with GraphCast consistently performing best in terms of RMSE, followed by Pangu‐Weather, and IFS. These models score very similarly, and their systematic differences are smaller than the uncertainty of the initialization. For the FSS, Pangu‐Weather and GraphCast are able to achieve similar scores to IFS, but do not consistently outperform it. This highlights how the RMSE, which is used in model training, is an incomplete perspective on model performance.

The perceived discrepancy between the case study and the evaluation of the North American convective season shows how case studies can be misleading for performance evaluation. The case study takes place in spring in the Southeastern United States, while the bulk of the convective season is driven by the Great Plains in summer. Particularly the moderately good performance of FourCastNet in this instance is not typical when over most of the summer season, RH and subsequently CAPE are largely overestimated. An additional example case during the summer is provided in Figure S3 in Supporting Information S1. This highlights the necessity to evaluate a larger sample of cases, before drawing conclusions on AI‐model‐performance. While a single convective season is still rather short, the consistency of the results across four different global regions indicates robustness in our evaluation.

Despite the shortcomings of deriving CAPE from vertically coarse pressure levels, the promising results of GraphCast and Pangu‐Weather indicate that CAPE is a worthwhile prediction variable. Predicting CAPE directly by training on ERA‐5's model‐level CAPE instead of deriving it, would likely be the most skillful approach. For instance, foundation models are designed for easier addition of variables after the main training of the model.

While forecasting convective environments is a large step closer to application‐oriented forecasting, it still lacks the specificity of impact‐oriented hazard predictions. Rapid progress in downscaling through super‐resolution may enable direct hazard forecasts in the foreseeable future.

Currently, the vertical coarseness limits the number of convectively relevant variables that can be derived. We refrained from evaluating convective inhibition, near‐surface shear, or helicity due to the limited resolution. However, these are key metrics for assessing convective initiation and convective hazards, especially tornadoes. With forecasters exploiting a myriad of convective parameters, it remains to be seen how practicable the direct prediction of additional convective parameters is in contrast to increasing the vertical resolution.

Taking this first step of deriving CAPE and DLS in AI‐models already provides useful information for severe convective outlooks, which are key for severe weather warnings. This highlights how application‐oriented products can be derived from AI‐generated forecasts, with similar skill to operational numerical models. Especially with their very short runtime, they can deliver very timely forecasts at a low computational cost. This step toward process‐based assessment of AI‐models is crucial for the future development of hazard‐driven applications.

## Supporting information

Supporting Information S1

## Data Availability

The IFS, ERA‐5, Pangu‐Weather, and GraphCast data were accessed through “Weatherbench2” (Google, LLC, 2023; Rasp et al., 2024). FourCastNet predictions were computed with the code release of ECMWF (ECMWF, 2023). The code used to process the forecast data and produce the visualizations can be accessed at https://github.com/feldmann‐m/AI‐storm with the assigned DOI https://doi.org/10.5281/zenodo.12527391 (Feldmann, 2024).
