# Supplementary material for: Lightning‐Fast Convective Outlooks: Predicting Severe Convective Environments With Global AI‐Based Weather Models
Source: Geophys Res Lett. 2024 Nov 21;51(22):e2024GL110960. doi: 10.1029/2024GL110960 (PMC11579977; doi:10.1029/2024GL110960)
Supplement: Supplementary file 1 — Supporting Information S1 [file GRL-51-0-s001.pdf]

Supporting Information for "Lightning-Fast Convective Outlooks: Predicting Severe Convective Environments with Global AI-based Weather Models"

Monika Feldmann[1], Tom Beucler[2], Milton Gomez[2], Olivia Martius[1]

[1]Institute of Geography - Oeschger Centre for Climate Change Research, University of Bern

[2]Faculty of Geosciences and Environment - Expertise Center for Climate Extremes, University of Lausanne

Contents of this file

1. Text S1: Computation of convective parameters
2. Table S1: Model Information
3. Table S2: Performance Scores
4. Table S3: Convective Regions
5. Table S4: Forecast scores of April 13, 06 UTC, 30h lead-time
6. Table S5: Forecast scores of April 13, 06 UTC, 74 lead-time
7. Figure S1: Comparison of vertical profiles for April 12, 2020, 12 UTC
8. Figure S2: 12 UTC initializations for seasonal scores in the USA

Corresponding author: M. Feldmann (monika.feldmann@unibe.ch)

9. Figure S3: CAPE and DLS forecasts for July 7, 2020, 06 UTC

Introduction

The supporting information presented here contains additional details in the computation of convective parameters, the interpretation of performance scores, an additional case study, and performance scores for the variable WMS.

Text S1: Computation of convective parameters

To compute the most unstable CAPE, the parcel profile is launched at the level of the highest equivalent potential temperature. WRF-python takes the surface temperature, pressure, and altitude as input, in addition to the pressure-level temperature, humidity, and geopotential height. Thus it accounts for the altitude of the surface for parcel profiles. CAPE itself is the integral between the parcel and environmental temperature profiles, where the parcel is buoyant.

$$CAPE = g \cdot \int_{z_{LFC}}^{z_{LNB}} \frac{T'_v - T_v}{T_v} \, dz \,, \tag{1}$$

where T_v is the virtual temperature of the environment, T'_v the virtual temperature of the lifted parcel, g the gravity constant, z the altitude, LFC the level of free convection and LNB the level of neutral buoyancy.

To obtain the DLS, the magnitude of the differential vector between surface and mid-atmospheric winds is computed:

$$DLS = \sqrt{(u_{500} - u_{10m})^2 + (v_{500} - v_{10m})^2} \,, \tag{2}$$

where u and v are the horizontal components of wind at the surface and 500 hPa level. The compound parameter WMS combines DLS and CAPE in the following manner:

$$WMS = \sqrt{2 \cdot CAPE} \cdot DLS \tag{3}$$

Table S1: Model information

Table S1 provides an overview of the available pressure levels for each model, their primary moisture variable, and the main training period.

Table S2: Performance Scores

Table S2 summarizes the range of each forecast score, as well as its optimal value. The FSS identifies the area fraction of the target variable exceeding a user-defined threshold. We use an area with a radius of 1° in which the fraction exceeding the threshold is evaluated. A FSS value of 1 indicates a perfect forecast (see Table S2). To interpret the SAL, a negative structure score indicates a too-fine spatial structure and too-small areas; a positive score indicates a too-large and too-flat distribution of values (smoothing). A low amplitude score indicates an underestimation, and a high score an overestimation. The location score accounts for both a mismatch in centroid, as well as a mismatch in overlap. A value of zero in each component indicates are perfect forecast (see Table S2).

Table S3: Convective Regions

Table S3 indicates the area used for each region, as well as the months of their respective convective seasons.

Table S4: Forecast scores of April 12, 12 UTC, 12h lead-time The scores for both Tables S4 and S5 are computed including the sea area, and exclusively in the domain shown in Figs 1 and 2 of the main manuscript.

Table S5: Forecast scores of April 12, 12 UTC, 156h lead-time

Figure S1: Comparison of vertical profiles for April 12, 2020, 12 UTC

Figure S2: 12 UTC initializations for seasonal scores in the USA

Figure S3: CAPE and DLS forecasts for July 7, 2020, 06 UTC

Model	Pressure levels	Moisture variable	Training period
IFS	50, 100, 150, 200, 250, 300, 400, 500, 600, 700, 850, 925, 1000	Q	
ERA5	50, 100, 150, 200, 250, 300, 400, 500, 600, 700, 850, 925, 1000	Q	
Pangu-Weather*	50, 100, 150, 200, 250, 300, 400, 500, 600, 700, 850, 925, 1000	Q	1979-2017
GraphCast*	1, 2, 3, 5, 7, 10, 20, 30, 50, 70, 100, 125, 150, 175, 200, 225, 250, 300, 350, 400, 450, 500, 550, 600, 650, 700, 750, 775, 800, 825, 850, 875, 900, 925, 950, 975, 1000	Q	1979-2017
GraphCast-oper*	50, 100, 150, 200, 250, 300, 400, 500, 600, 700, 850, 925, 1000	Q	1979-2017
FourCastNet*	50, 100, 150, 200, 250, 300, 400, 500, 600, 700, 850, 925, 1000	RH	1979-2015
	Surface variables		
all datasets	2m temperature, 10m wind, mean sea level pressure		

Table S1. Model characteristics, neural weather models marked with an asterisk

Score	Value range	Optimal score
RMSE	≥ 0	0
BIAS	$\pm\infty$	0
FSS	0,1	1
S	-2,2	0
A	-2,2	0
L	0,2	0

Table S2. Forecast evaluation metrics

Region	Latitudes	Longitudes	Months
North America	20/50	250/300	April-August
Europe	35/55	350/30	April-September
Argentina	-40/-25	290/310	September-February
Australia	-35/-20	140/155	September-February

Table S3. Regions

Score	IFS	Pangu-Weather-oper	GraphCast-oper	FourCastNet-oper
		CAPE		
RMSE	312	286	***271**	386
BIAS	-88	90	***1**	-92
FSS_{300}	0.92	0.93	***0.96**	0.93
FSS_{1000}	0.87	***0.91**	*0.88	0.61
S	0.21	0.23	***0.02**	0.27
A	0.18	0.16	***-0.04**	-0.28
L	0.03	***0.02**	0.02	0.03
		DLS		
RMSE	3.3	3.3	***2.9**	3.5
BIAS	0.2	0.2	-0.2	***0.1**
		WMS		
RMSE	332	303	***262**	334
BIAS	88	90	***-24**	-39
FSS_{300}	0.91	0.93	***0.96**	0.92
FSS_{500}	0.91	0.93	***0.95**	0.92
S	*0.31	0.29	0.11	**0.03**
A	0.18	0.18	***-0.06**	-0.09
L	0.03	0.05	***0.02**	*0.03

Table S4. Forecast scores at 12h lead-time, best model is marked in bold and with an asterisk

Score	IFS	Pangu-Weather-oper	GraphCast-oper	FourCastNet-oper
CAPE				
RMSE	829	570	***520**	606
BIAS	-223	210	***60**	-288
FSS_{300}	0.26	0.76	***0.77**	0.51
FSS_{1000}	0.20	0.71	***0.74**	0.36
S	0.54	0.39	***0.17**	0.63
A	-0.69	0.35	***0.09**	-1.06
L	0.34	0.11	***0.09**	0.36
DLS				
RMSE	10.2	***5.1**	5.1	7.7
BIAS	3.2	1.2	***-0.2**	1.2
WMS				
RMSE	870	577	***511**	604
BIAS	-124	216	***39**	-257
FSS_{300}	0.26	0.76	***0.77**	0.51
FSS_{500}	0.26	0.71	***0.74**	0.36
S	0.33	0.51	***0.19**	0.36
A	-0.33	0.40	***0.08**	-0.84
L	0.35	0.11	***0.10**	0.37

Table S5. Same as Table S4 but for 156h lead-time

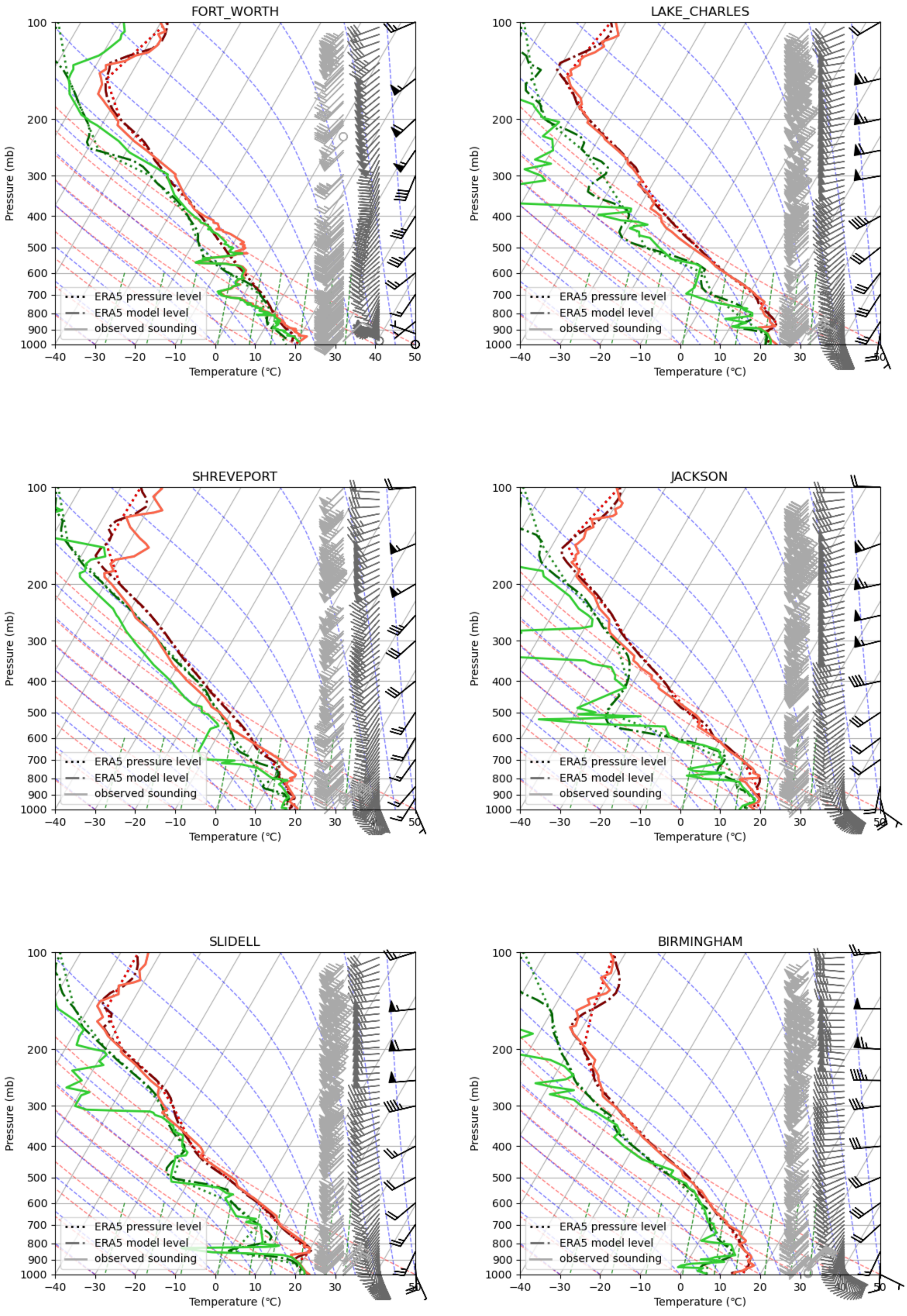

Figure S1. Comparison of soundings from ERA-5 pressure levels, model levels, and measured radiosoundings from 6 locations on April 12, 2020, 12 UTC

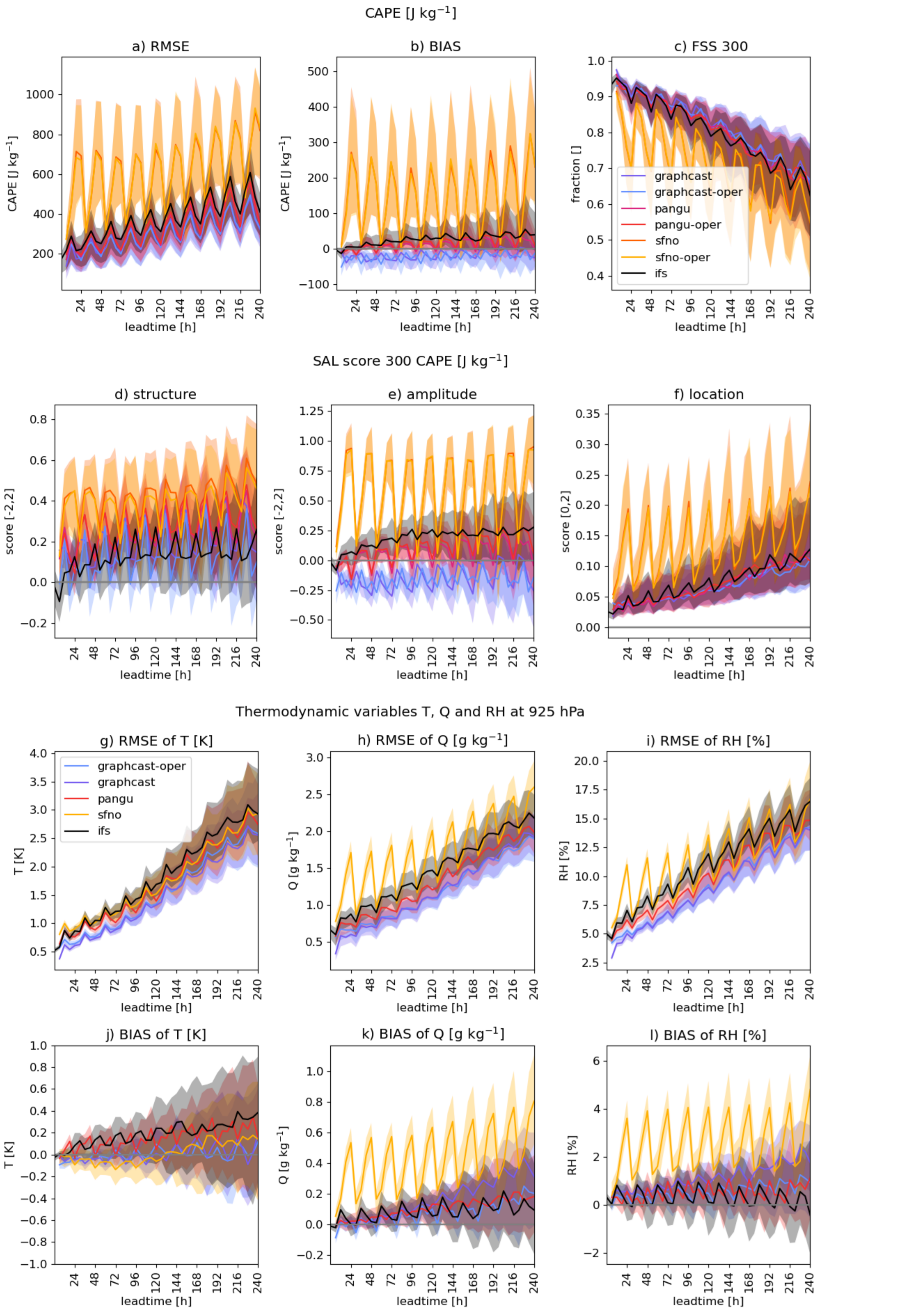

Figure S2. Seasonal evaluation of CAPE for North America at 12 UTC initialization shows Pangu-Weather and GraphCast performing better than IFS, with a moisture bias impacting the performance of FourCastNet. Top row: a) RMSE, b) BIAS, c) FSS_{300}; Second row: SAL components for CAPE >300 J kg^{-1}; color legend in panel c); Third row: RMSE of T, Q and RH at 925 hPa (g-i); Fourth row: BIAS (j-l); model legend in panel g). Solid line depicts the median and shading the interquartile range.

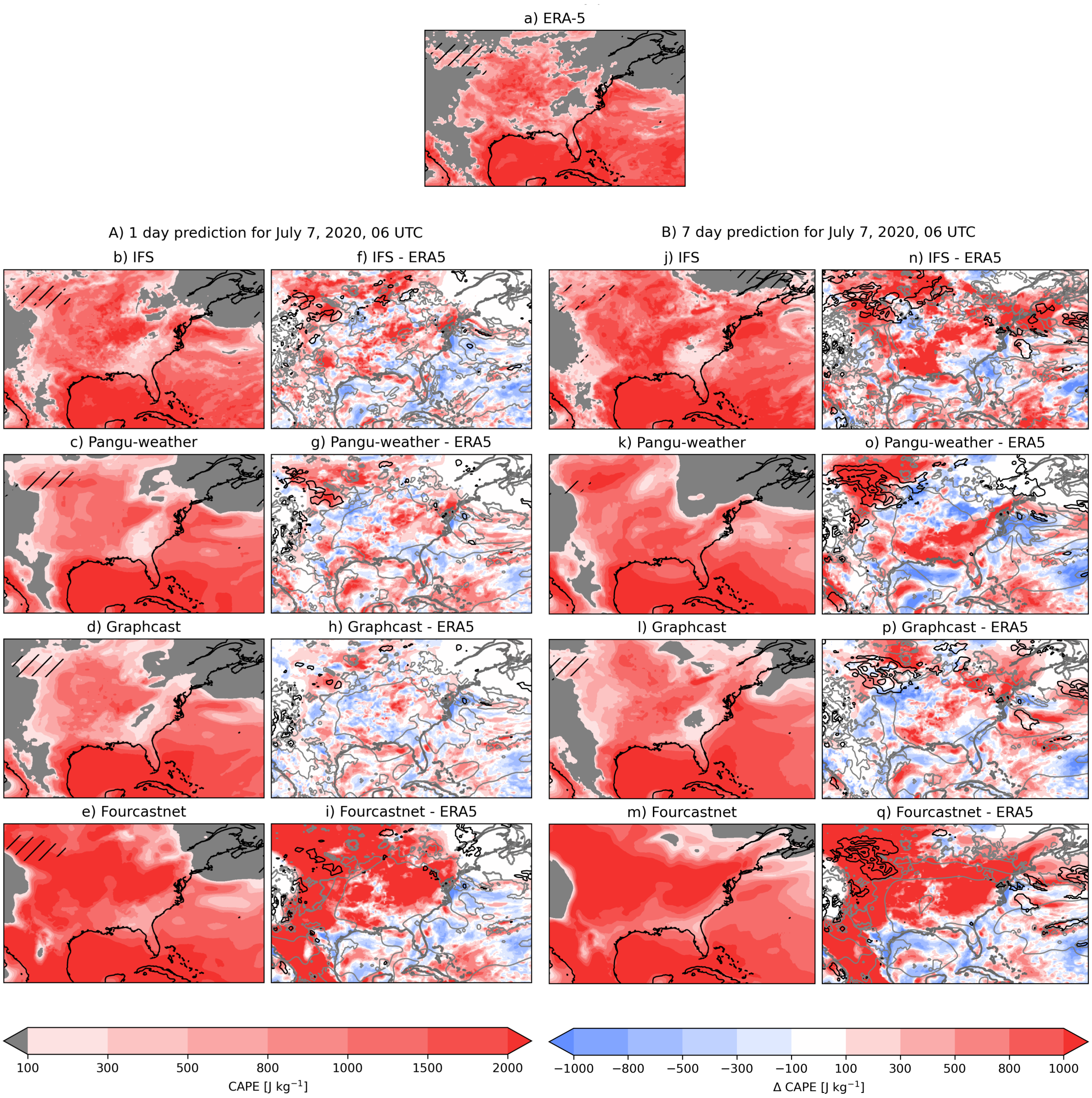

Figure S3. Comparison of forecasts of CAPE and DLS at 1 and 7 days lead-time; a) ERA-5 data of CAPE and DLS on July 7, 2020 at 06 UTC; A) 1 day forecast of CAPE and DLS (b-e) in comparison to ERA5 (f-i) B) 7 day forecast of CAPE and DLS (j-m) in comparison to ERA5 (n-q); hatched area indicates DLS >20 m s^{-1}; contours indicated positive (grey) and negative (black) areas of Δ DLS in 5 m s^{-1} increments
